# Stress distribution and fracture resistance of green reprocessed polyetheretherketone (PEEK) single implant crown restorations compared to unreprocessed PEEK and Zirconia: an in-vitro study

**DOI:** 10.1186/s12903-023-02943-x

**Published:** 2023-05-11

**Authors:** Marwa Emam, Ahmed Mohamed Arafa

**Affiliations:** 1 Fixed Prosthodontics Department, Faculty of Dentistry, Ain Shams University, Organization of African Unity St, El-Qobba Bridge, Al Waili, 11566 Cairo, Egypt; 2 Fixed Prosthodontics Department, Faculty of Dentistry, Beni-Suef University, Beni-Suef, Egypt

**Keywords:** Green dentistry, PEEK reprocessing, Zirconia, Dental implant, Heat pressing, Fracture resistance, Stress, Strain, Modulus of elasticity

## Abstract

**Background:**

It is unclear which crown materials are optimum to disperse the generated stresses around dental implants. The objective of this study is to assess stress distribution and fracture resistance of green reprocessed Polyetheretherketone (PEEK) in comparison to un-reprocessed PEEK and zirconia single implant crown restorations.

**Methods:**

Twenty crowns (n = 20) were obtained, five from zirconia and fifteen from pressed PEEK that were subdivided into 3 groups of five specimens each (n = 5) according to weight% of reprocessed material used. A 100% new PEEK was used for the first group, 50% new and 50% reprocessed PEEK were used for the second group, and a 100% reprocessed PEEK was used for the third group. Epoxy resin model with dental implant in the second mandibular premolar was constructed with strain gauges located mesially and distally to the implant to record strain while a load of 100 N was applied with 0.5 mm/min then specimens of all groups were vertically loaded till failure in a universal testing machine at cross head speed 1 mm/min. Data was statistically analyzed by using One-way Analysis of Variance (ANOVA) followed by Post-hoc test when ANOVA test is significant.

**Results:**

No significant difference between strain values of tested groups (*p* = 0.174) was noticed. However, a significant difference between fracture resistance values was noticed where the zirconia group recorded a significantly higher value (*p* < 0.001).

**Conclusions:**

Implant restorative materials with different moduli of elasticity have similar effects regarding stresses distributed through dental implant and their surrounding bone. Reprocessed PEEK implant restorations transmit similar stresses to dental implant and surrounding bone as non-reprocessed PEEK and zirconia restorations. Zirconia failed at higher load values than all tested PEEK restorations but all can be safely used in the posterior area as crown restorations for single implants.

**Clinical relevance:**

Applying “green dentistry” principles may extend to include reprocessing of pressed PEEK restorative materials without affecting the material’s shock absorption properties.

## Background

Dental implants have a high success rate for rehabilitation in patients with partial or complete dental loss with aesthetically pleasing and functional restorations [1]. One of the main causes of fractures and dental implant loss is masticatory overload. When prosthetic pieces made of materials with differing elastic modulus are used, these parts may cause the implant and peri-implant bone to experience distinct stresses and strains [2, 3]. Titanium abutments are the gold standard for implant rehabilitation, but the selection of the crown material is another crucial consideration [1]. A large variety of indirect esthetic materials exist to fabricate the restorations. Given the stiffness of these materials, the elastic modulus can range from that of zirconia to a polymeric material [4].

Due to their high aesthetics and outstanding biocompatibility, ceramic materials like zirconia (Zr) and glass ceramic have been employed extensively. However, these rigid materials may transmit excessive stresses to the implant-prosthesis complex, leading to biological as well as technical difficulties [5]. Recently, dental restorations made of high-performance polymers (HPPs) are becoming more and more common. Polyetheretherketone (PEEK) is a major representative of polyaryletherketone (PAEK) family. It is a thermoplastic substance that is highly effective, temperature resistant, and partially crystalline. It offers many advantages including stable physical qualities, biocompatibility, and good abrasion resistance, with low modulus of elasticity similar to the bone so it is preferred in dentistry because it provides stress breaking action reducing pressures imparted to implants and their supporting structures [6–8].

PEEK restorations are made using either pressing technology or computer-aided design, computer-aided manufacturing (CAD-CAM). The sprues and the residual button material should be cut after pressing and thrown away. For new pressings, fresh material ingots ought to be employed. However, this will result in a substantial amount of material being wasted [9].

As environmental responsibility grows more obvious, society prioritizes protecting the environment, and numerous organizations are promoting eco-friendly behavior. “Green dentistry,“ a high-tech approach, strives to decrease the impact of dental offices on the environment while also including a service delivery paradigm for dentistry that supports and sustains wellness [10].

Reusing waste is a green dentistry concept that promotes giving an item new life in order to prolong its usefulness and keep it from becoming waste. Reusing products relieves the strain on natural resources and lowers the energy required to create new ones [10]. Recycling is also a crucial component of waste management since it reduces resource consumption and the amount of waste dumped in landfills [10]. In previous studies, industrial PEEK has been recycled and the effect on its mechanical properties has been investigated [11–15]. But the PEEK composition used for dental applications and reprocessing effects on its shock absorption properties has not been addressed.

It is unclear which crown materials are optimum to disperse the generated stresses and thereby assure a higher lifespan. The present study aimed to assess the stress distribution on implant supporting structures and fracture resistance of PEEK conditions (new, partially or totally reprocessed) and zirconia as single implant crown restorative material. The null hypothesis was that neither stress distribution nor fracture resistance would be affected by the material of single implant crown restorations or by increasing the percentage of reprocessed PEEK.

## Methods

In this study, the stress distribution and fracture resistance of PEEK and zirconia single implant crown dental restorations were tested. A power analysis was determined based on the results of a previous study [16]. By adopting (0.05) alpha (α) level, (0.05) beta (β), (power = 95%), and (3.344) effect size (f); the predicted minimum sample size (n) was a total of twelve (3 samples per group). G*Power version 3.1.9.7 was used for sample size calculation [17].

Twenty crowns (n = 20) were fabricated, five from zirconia (inCoris TZI C medi S A1 (Sirona Dental Systems, Fabrikstrasse 31 D-64,625 Bensheim, Germany) and fifteen from pressed PEEK (for 2 press BioHPP Granulate, Bredent GmbH & Co KG, senden, Germany) that were subdivided according to the weight% of new and reprocessed PEEK material used into three groups of five specimens each (n = 5). The first group consisted entirely of new PEEK, the second group of partially reprocessed PEEK composed of a 50/50 ratio of new and reprocessed PEEK, and the third group of totally reprocessed PEEK.

### Master dies preparation

**Fig. 1:**
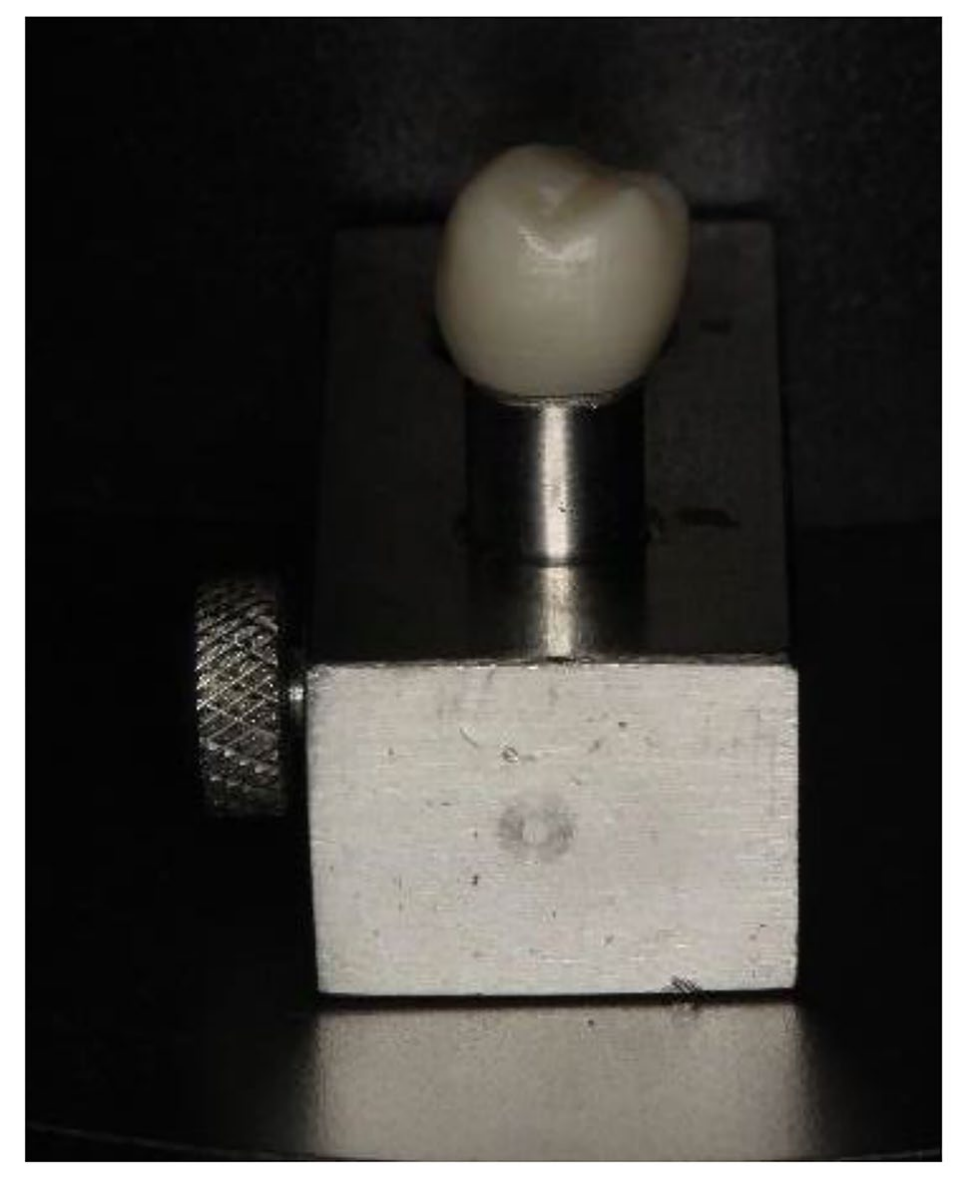
Zirconia crown on stainless-steel die fixed in holder with a screw

A stainless-steel die simulating a prepared second lower premolar was designed to receive a single crown restoration. The die was machine milled to 5 mm height, 6 mm diameter, flat occlusal surface, total occlusal convergence angle of 12^°^, 1 mm chamfer finish line, and was fixed in a stainless-steel holder with a screw (Fig. 1).

### Fabrication of epoxy resin cast with implant

A mandibular model (Kilgore Int. Inc., cold water, mish., USA) used for educational purposes which accurately replicates the anatomical details of the teeth and surrounding structures was used. The mandibular posterior teeth in one quadrant were removed to create an edentulous span for the implant and the strain gauges, and the socket was sealed with wax. One tapered screw vent implant (Zimmer Dental, California, USA) 3.5 × 13 mm with an impression coping was attached to the socket in the position of the lower second premolar by sticky wax. After complete hardening of the wax, alignment, angulations as well as stability of the implant were checked. After impression taking, the implant was released from the model then relocated to its corresponding position in the impression.

Self-cured epoxy resin model (System Three Resins, Inc. 3500 W. Valley Hwy N; Suite 105 Auburn, WA 98,001 − 2436, USA) was poured in the impression to replicate the alveolar bone for the implant insertion and surrounding dental structures. The implant with the scan body attached and stainless-steel die after spraying with Cerec Optispray (Sirona Dental Systems GmbH, Bensheim, Germany) were scanned separately. The STL files of both scans were imported to CAD design software (Exocad Dental CAD; exocad GmbH, Germany). The implant abutment was designed following the same dimensions of the die. The STL file of designed abutment was saved and sent to mill a custom-made titanium implant abutment with internal hex from Ti–6Al–4 V alloy discs (Starbond Ti5 Disc, Scheftner, Mainz, Germany) which was later screwed to the implant in the resin model.

### Fabrication of Zirconia restorations by milling technology

Cerec inLab (Sirona Dental Systems GmbH, Germany) was employed to fabricate the zirconia restorations. It consisted of a Personal Computer for Cerec inLab 3D software (version 4.2), inEos Desktop Blue scanner, and MC XL milling unit. The stainless-steel die was scanned with InEos X5 Desktop scanner after spraying with Cerec Optispray to obtain the best scanning accuracy. Cerec software was used to form a virtual 3D model that was used to design a full anatomical crown with axial dimensions of 1.5 mm, and occlusal thickness of 2 mm. Monolithic crowns were milled by Cerec inLab MC XL milling unit from inCoris TZI A1blocks (Sirona Dental Systems GmbH, Germany).

After milling, zirconia restorations were separated and steamed off to prevent any milling residues from remaining in the fissures. Sintering was performed following the manufacturer’s recommendation using a special furnace (inFire HTC, Sirona Dental Systems GmbH, Germany) by using the preset program of inCoris ZI. The heating rate and duration time is demonstrated in (Table 1).

**Table 1:** Sintering parameters for the Zirconia specimens:

Heating rate (ºC /min)	Holding temperature °C	Holding time (min)
25	800	0
15	1510	120
30	200	0

The sintered zirconia crowns were then examined for deformation and debris before being cleaned with steam. Each zirconia crown was seated on the stainless-steel die and checked for passive fit before cleaning for 10 min in an ultrasonic cleaner (Silfradent, S.Sofia, Forl, Italy). Finally, the zirconia crowns were polished using CEREC advanced polishing kit (Hager and Meisinger GmbH, Germany) following the manufacturer’s recommendations using polishing cups, discs and points of varying grit sizes, starting with the green rubber polishing tips followed by blue rubber polishing tips and finally, red rubber tips used to give a “wet” look. The duration of polishing is 30 s per polishing level using a rotary speed of 10.000 rpm without water or spray cooling. After completion of polishing, the zirconia crowns were rinsed and dried.

### Construction of resin patterns

Based on the same design used for zirconia crowns, 10 resin patterns were milled out of polymethyl methacrylate blocks (PMMA disk; Yamahachi, Dental Mfg, Co) by the same milling unit to be pressed later into PEEK restorations.

### Fabrication of PEEK restorations by pressing technology

The milled resin patterns were transformed into PEEK by lost wax and heat pressing procedures. Phosphate-bonded investment material (Brevest For2press, Bredent, Germany) was used to invest the sprued resin patterns following the manufacturer’s instructions. After complete setting, the muffle and the disposable extrusion punch were placed in the preheated furnace for 60 min/630–850 °C. Subsequently, the temperature was slowly lowered (max 8 °C/minute) to the necessary 400 °C melting temperature of the BioHPP for2press material and kept for 60 min. The muffle was then filled according to PEEK conditions, 100% brand-new PEEK made up the first group. A new and reprocessed PEEK mix of 50% each was used for the partially reprocessed PEEK second group. 100% reprocessed PEEK was used for the third group depending on the wax weight of the model. After complete melting, PEEK granules were pressed into the muffle with the preheated extrusion punch using the press device (For2press, GmbH & Co KG, senden, Germany). After the cooling, The muffle was removed from the device and the crowns were divested using a blasting device (Basic Classic, Renfert GmbH, Germany) utilizing 110 μm alumina particles at 3 bar pressure. Finishing and polishing of the PEEK crowns were performed in accordance with the manufacturer’s instructions using a rubber polisher followed by high-gloss polishing paste (Abraso-Starglanz, Bredent, Gmbh, Germany).

### Strain analysis measurements

**Fig. 2:**
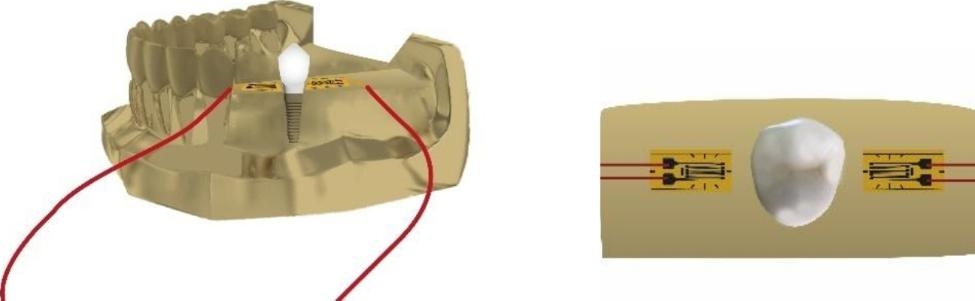
Schematic diagram of the epoxy resin model with strain gauge attached

**Fig. 3:**
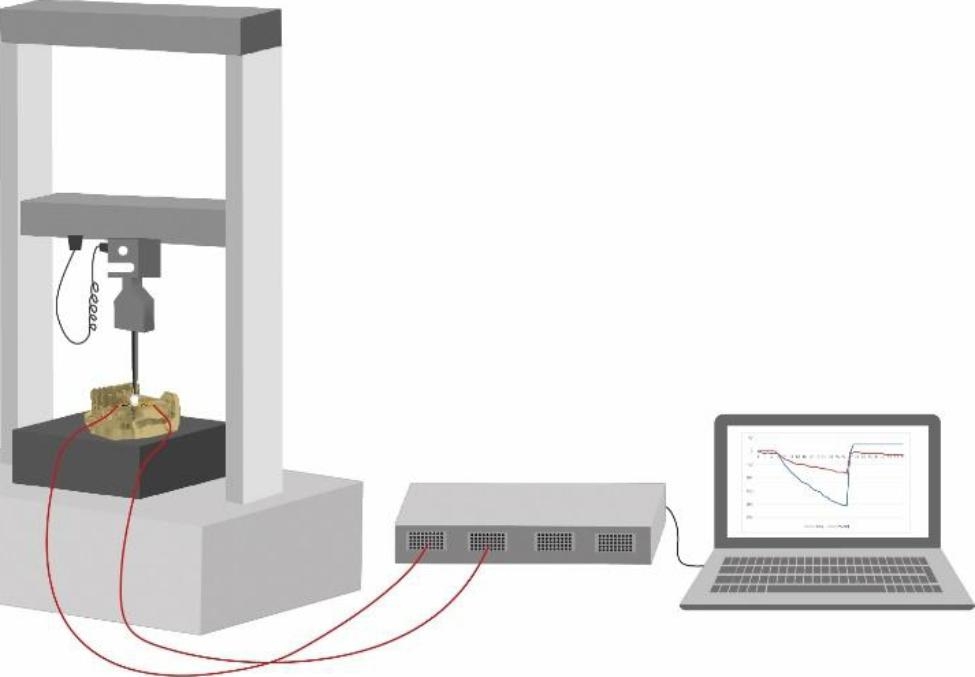
Schematic diagram of loading the implant restoration in the resin model and collecting strain data

Strain gauges were bonded to the mesial and distal surfaces of the epoxy resin model around the implant (Fig. 2). The two strain gauges were connected to different channels of the strain meter which was connected to a compatible computer running the meter control software (PCD 300 A, Kyowa-Electronic Instruments Co, LTD, Tokyo, Japan). Prior to performing the test, the meter was balanced to zero. The model was secured to the lower compartment of a universal testing machine with a load cell of 5 KN. The specimens of all groups were slowly loaded with an increasing vertical load of 100 N with 0.5 mm/min while simultaneously recording strain data. (Fig. 3).

The measurements were carried out together with specialized software to analyze the strain that occurred.

### Fracture resistance measurements

All specimens of tested groups were loaded compressively in the universal testing machine at a cross head speed of 1 mm/min [18]. Each specimen was individually placed on the stainless-steel die secured by the holder. A vertically moveable rod with a 5 mm-diameter semi-spherical head was positioned directly over the occlusal surface in the central fossa after the placement of a piece of tin foil between the load piston and the specimen to achieve an even stress distribution. The fracture resistance value or failure load was determined with the value accompanying the first crack in the loaded specimen. The load at failure was recorded in Newton as soon as load decreased by 10% of the maximum load.

### Statistical analysis

Data was presented as mean, standard deviation (SD), median and interquartile range (IQR) values. Shapiro-Wilk’s test was used to check for normality and homogeneity of variances was checked using Levene’s test. Data was analyzed using one-way ANOVA followed by Tukey’s post hoc test where the significance level was set at *p* < 0.05. Statistical analysis was performed with R statistical analysis software version 4.1.3 for Windows^1^.

## Results

Descriptive statistics for strain and fracture resistance values as well as results of intergroup comparisons are presented in Table 2. Regarding strain values, no significant difference between tested groups (*p* = 0.174) was noticed. However, a comparison between fracture resistance values showed a significant difference with the zirconia group recording a significantly higher value (*p* < 0.001).

**Table 2:** Descriptive statistics and intergroup comparison

Measurement	Group	Mean	SD	95% CI		Median	IQR	f-value	p-value	
				Lower	Upper					
**Strain (µm/m)**	**New PEEK**	148.13^ A^	8.35	140.81	155.46	147.50	9.33	1.88	**0.174**	
	**Partially reprocessed PEEK**	163.33^ A^	10.34	154.27	172.40	159.17	11.67			
	**Totally reprocessed PEEK**	162.50^ A^	8.31	155.21	169.79	165.83	12.50			
	**Zirconia**	142.83^ A^	29.71	116.79	168.87	126.67	50.00			
**Fracture resistance (N)**	**New PEEK**	1388.93^B^	238.46	1179.91	1597.94	1368.97	345.49	85.70	< 0.001*	
	**Partially reprocessed PEEK**	1203.92^B^	99.23	1116.94	1290.89	1162.66	186.66			
	**Totally reprocessed PEEK**	1157.39^B^	346.69	853.51	1461.28	1059.41	335.43			
	**Zirconia**	4333.92^ A^	608.51	3800.55	4867.30	4591.67	825.01			

95%CI = 95% confidence interval for the mean; SD = standard deviation; IQR = interquartile range

Dissimilar superscript letters indicate significant difference.

## Discussion

The stresses in implant-supported fixed dental prostheses come from functional forces that the restorative material, abutment, and implant convey to the supporting bone. Extreme stress concentrations should be reduced, and the stressors must be at physiological levels. That’s why it is necessary to assess the stresses in the materials and supporting tissues [19]. When loading an implant restored with a stiff occlusal material, like metal or zirconia, the implant, and the supporting bone may experience considerable impulse loading. Resins and polymers with low modulus of elasticity were claimed to reduce strains on the implants and the osseous system that supports them [3, 19]. Many studies have recommended PEEK as an alternative option to fabricate single crowns and fixed dental prostheses (FDPs) [20–24]. but limited studies on the impact of restorative material type on distributed stress in implant-supporting structures are available.

Green dentistry is a comprehensive strategy of dental care that lessens its negative effects on the environment while fostering a compassionate atmosphere for patients [10]. By application of the four R’s model “Rethink, Reduce, Reuse and Recycle” [10]. Not only materials like paper cups, paper, magazines, general waste, and clothes in the dentist’s clinic can be recycled, but the concept should also expand to include all materials that can be reprocessed without losing their properties including restorative materials. This should also help reduce costs as well as waste [25]. To the knowledge of the authors, only one study evaluated the impact of PEEK reprocessing on fracture resistance of dental restorations [18].

The present study evaluated the stress distribution on implant supporting structures and fracture resistance of PEEK conditions (new, partially or totally reprocessed) and zirconia as single implant crown restorative material.

As a result of the analysis, The zirconia group revealed a significantly higher fracture resistance than other PEEK groups while an insignificant difference in strain values was noticed between all tested groups so the null hypothesis stated that neither stress distribution nor fracture resistance would be affected by the material of single implant crown restorations or by increasing the percentage of reprocessed PEEK was partially accepted.

Hooke’s law affirms that less force is delivered to the implant and supporting bony structures due to the reduced stiffness and higher elastic deformation of the implant restorative material that results from its lower modulus of elasticity [26]. In the present study, the insignificant difference between strain values recorded on the bone surface around dental implants was induced by different restorative materials. This is consistent with previous studies [27–31]. Datte et al. [1] and Sevimay et al. [19] are finite element studies that evaluated the effect of different restorative materials on bone strain in response to an applied load of 200 and 300 N respectively in finite element studies. It was found that different materials didn’t have any effect on the bone strain. Moreover, the recorded stresses on prosthetic parts and supporting bone were less than the components’ ultimate and yield strength values induced by different restorative materials. The first study used cemented crowns without a mention of its type while the second neglected the cement layer while creating the 3D model. Besides, Isidor [32] failed to find evidence of damaging effects on the bone tissue induced by zirconia restorative material on implants. However, plastic materials might lack the abrasion resistance necessary for solid occlusal contact over prolonged usage [33].

Similarly, it was found by Ausiello et al. [34] that under a load of 600 N, the 100 μm resin cement layer and the crown representing lower molar were the only structures where the variation in the crown’s material had an impact. Differences in the crown elastic modulus had no impact on the other structures beneath them. As the overall stiffness of the models is equivalent, except for the crown material, the stress distributions have been the same whenever going away from the prosthetic complex zone. Thus, stresses in the supporting bone were not affected [31].

Alternatively, Eskitascioglu et al. [35] and Benzing et al. [36] concluded that superstructures made of low elastic modulus alloys would result in higher strains at the bone-implant contact than matching prostheses made of rigid alloys. The same was found by Ahmed et al. [16] who studied the effect of different crown and abutment materials combinations on biomechanical analysis. Custom made abutments luted to Ti bases and crowns luted to underlying abutments were among their methodological steps. They concluded that zirconia crowns and abutment group had higher mean strain values around the implant than PEEK abutment & zirconia crown group, with the difference being statistically significant. The different results might be attributed to different restoration designs with custom abutments and crowns rather than metal abutments in our study.

Regarding fracture resistance, although the zirconia group revealed significantly higher values than other PEEK groups, all recorded values were above the maximum occlusal loads in the posterior region (up to 900 N) [37, 38]. This is in agreement with Tartuk et al. [39] who tested uncemented crowns made of zirconia, hybrid ceramics, and PEEK on a zirconia base model for load-bearing properties. Zirconia failed at significantly higher loads compared to the other materials. El Sokkary et al. [40] in their study followed up 24 full coverage restorations of PEEK and zirconia in the posterior region and found both materials to have a 100% survival rate after one year in the oral cavity without cracks or fractures. According to Türksayar et al. [41] who evaluated the fracture resistance of zirconia, reinforced polyetheretherketone (PEEK), and polyetherketoneketone (PEKK) implant abutments and glass ceramic crowns, the reinforced PEEK abutments showed a similar level of fracture resistance to the Zirconia abutments after thermomechanical aging. Additionally, Preis et al. [38] studied the fracture resistance of bonded implant-supported PEEK frameworks subjected to a static loading after chewing simulation and thermal cycling. The mean fracture load for zirconia was significantly higher than composite veneered PEEK restorations.

On the other hand, Saravi et al. [42] concluded that PEEK abutments showed superior load-bearing properties compared to zirconia abutments. PEEK had a mean fracture load of 1101 N, while zirconia reached 772 N. The difference was attributed to the difference in elastic modulus and the force-diverting properties of the PEEK material. The contradictory results might be due to different restoration designs. As the abutments were mimicking maxillary central implant restoration with cobalt chromium crowns and loading the samples was with 30^°^ angle rather than axial loading in our study.

Regarding PEEK reprocessing, a previous study [18] assessed the influence of reprocessing on the fracture resistance and the mode of failure of both new and reprocessed 3-unit FDPs PEEK, either partially or completely. They concluded that 3-unit FDPs made of entirely reprocessed PEEK recorded the least fracture resistance values between tested groups. However, all of them failed at loads higher than the maximal biting forces in the molar area. According to their research, adding recycled PEEK material during the pressing of 3-unit FDPs made them more brittle and reduced their capacity to deform under load. It was assumed that the cushioning effect of the PEEK material would be affected by reprocessing which was not proven in the present study. This might be due to the difference in restoration design as in FDPs, the load applied is concentrated in the connector area while in the present study, crowns allowed load distribution from the occlusal surface to axial walls.

Reprocessing PEEK provides financial and environmental advantages and although our study showed favorable encouraging results for reprocessing PEEK, it can not be applied clinically before studying the effect on other principal properties of the material such as surface roughness and bond strength to veneering material and dentin. Elastic modulus, homogeneity and other basic physical properties of reprocessed PEEK were not investigated as well and shall be addressed in future research.

All tested specimens in the present study were not cemented and not aged, which is considered a limitation of this study so further investigations with cyclic loading or thermocycling are required.

## Conclusions

Within this study’s limitations, these conclusions can be drawn:

1. Implant restorative materials with different moduli of elasticity have similar effects regarding stresses distributed through dental implant and their surrounding bone.
2. Reprocessed PEEK implant restorations transmit similar stresses to dental implant and surrounding bone as non-reprocessed PEEK and zirconia restorations.
3. Zirconia failed at higher load values than all tested PEEK restorations but all can be safely used in the posterior area as crown restorations for single implants.

## Data Availability

Datasets analyzed during the current study are available from the corresponding author on reasonable request.

## Footnotes

1 R Core Team (2022). R: A language and environment for statistical computing. R Foundation for Statistical Computing, Vienna, Austria. URL https://www.R-project.org/.
